# Mobile phones are a viable option for surveying young Australian women: a comparison of two telephone survey methods

**DOI:** 10.1186/1471-2288-11-159

**Published:** 2011-11-24

**Authors:** Bette Liu, Julia ML Brotherton, David Shellard, Basil Donovan, Marion Saville, John M Kaldor

**Affiliations:** 1 The Kirby Institute, University of New South Wales, Sydney, Australia; 2 Victorian Cytology Service, Melbourne, Australia; 3 The Hunter Valley Research Foundation, Newcastle, Australia; 4 Sydney Sexual Health Centre, Sydney Hospital, Sydney, Australia

**Keywords:** Cellular phone, mobile phone, telephone surveys, survey methods, HPV vaccine

## Abstract

**Background:**

Households with fixed-line telephones have decreased while mobile (cell) phone ownership has increased. We therefore sought to examine the feasibility of recruiting young women for a national health survey through random digit dialling mobile phones.

**Methods:**

Two samples of women aged 18 to 39 years were surveyed by random digit dialling fixed and mobile numbers. We compared participation rates and responses to a questionnaire between women surveyed by each contact method.

**Results:**

After dialling 5,390 fixed-lines and 3,697 mobile numbers, 140 and 128 women were recruited respectively. Among women contacted and found to be eligible, participation rates were 74% for fixed-lines and 88% for mobiles. Taking into account calls to numbers where eligibility was unknown (e.g. unanswered calls) the estimated response rates were 54% and 45% respectively. Of women contacted by fixed-line, 97% reported having a mobile while 61% of those contacted by mobile reported having a fixed-line at home. After adjusting for age, there were no significant differences between mobile-only and fixed-line responders with respect to education, residence, and various health behaviours; however compared to those with fixed-lines, mobile-only women were more likely to identify as Indigenous (OR 4.99, 95%CI 1.52-16.34) and less likely to live at home with their parents (OR 0.09, 95%CI 0.03-0.29).

**Conclusions:**

Random digit dialling mobile phones to conduct a health survey in young Australian women is feasible, gives a comparable response rate and a more representative sample than dialling fixed-lines only. Telephone surveys of young women should include mobile dialling.

## Background

Random digit dialling fixed telephone lines (landlines) is commonly used by health researchers to access a representative sample of the population for survey purposes. In Australia, given the large geographic distances and the high coverage of fixed-lines, it has been the method of choice [1,2]. However in recent years, in line with international trends, there has been a gradual decrease in the number of households with fixed-line telephones, particularly among young adults [3-6]. This has led to concerns regarding the representativeness of populations sampled by this method [7]. In parallel there has been a dramatic increase in the ownership and use of mobile phones,[3,6] yet despite this shift, there is very limited information on random digit dialling mobile phones to contact participants for population surveys [5]. To inform the design of a national survey of reproductive health in young women, we conducted a pilot study examining the feasibility of random digit dialling mobile phones and compared characteristics of women surveyed by this method with a separate sample of women surveyed by random digit dialling fixed-lines.

## Methods

We recruited two groups, each consisting of at least 100 women, by random digit dialling fixed-line household numbers and mobile numbers. Women were eligible for the survey if they were aged 18-39 years and able to communicate verbally in English. Telephone numbers were randomly generated and dialled. If a fixed-line was answered, the respondent was asked whether there was an eligible woman in the household, and if so, she was invited to participate in the survey. For mobiles, if the call was answered, and the recipient was an eligible woman, they were asked to take part. The survey was 5-10 minutes long (for full questionnaire see additional file 1) and included questions on human papillomavirus (HPV) immunisation, past diagnoses of genital warts and chlamydia testing and treatment. The survey also sought information on whether the participant had a mobile phone if recruited by fixed-line, or if they had a fixed-line telephone at home if recruited by mobile.

For households contacted by fixed-line, preference was given to women aged 21-39 years over those aged 18-20, and if there were still more than one, random selection was used. Sampling was stratified by age, to recruit women 21-30 years old and 31-39 years in a ratio of 3:1, and state of residence, to recruit women in similar proportions to the geographic distribution of young women in Australia. Age stratification was required to ensure adequate power for pre-specified comparisons in the larger study. There was no pre-defined quota for women aged 18-20 years and they were included if their number was randomly dialled and no women aged 21-39 were available.

The selection process for fixed-line and mobile numbers was similar. Lists of currently active numbers were selected from the White Pages telephone book and modified by random terminal digit substitution to generate sets of numbers with proportional national distributions of number prefixes. Following number selection, a maximum of six calls were made to establish contact and a maximum of five more to complete the survey if there was an eligible person at the number. The survey was conducted using a standardised script and a Computer Assisted Telephone Interview (CATI) program. The outcome of every number called and responses to all survey questions were recorded. Both groups were surveyed by trained interviewers.

We compared call and survey responses between the two contact methods. Each number dialled was categorised as either 'eligible' (contact was in the target sex and age group), 'ineligible' (contact was incorrect sex or age, or had difficulty with English), 'unknown eligible' (answering machine/voicemail, no answer, refusal before eligibility determined) or 'invalid' (business, fax, disconnected). Two call response rates were calculated by dividing the number of interviews completed by: i) the number of 'eligible' calls; and ii) the number of 'eligible' calls plus a proportion of the 'unknown eligible' calls estimated to be eligible. The proportion of 'unknown eligible calls' estimated to be eligible was derived by using the population where eligibility was known and dividing all 'eligible' calls by all 'eligible' and 'ineligible' calls. For each main survey question we compared the response between the two groups (fixed-line and mobile) using logistic regression, adjusted for participant age. The study was approved by the University of New South Wales Human Research Ethics Committee.

## Results

Throughout March 2011, 5,390 randomly selected fixed-line numbers and 3,697 mobile numbers were called. Of these, 2.6% (140/5390) of the fixed-line and 3.5% (128/3697) of the mobile numbers resulted in an eligible woman being surveyed. For most numbers called (see Table 1) there were either no eligible respondents (36% of fixed-line; 33% of mobile calls) or the number was invalid (46% fixed-line; 28% mobile). A substantially greater proportion of calls made to mobiles resulted in a message being left on a recording device, with no subsequent response being received on the study free call return number (26% mobile; 6% fixed-line). Of calls where an individual was determined to be eligible, the questionnaire completion rates were 74% and 88% for fixed-line and mobiles respectively (p = 0.1 for difference in proportions). Based on the estimated proportion of 'unknown eligible' numbers thought to be eligible, response rates fell to 54% and 45% respectively (p = 0.04 for difference in proportions).

**Table 1:** Call outcomes for all numbers dialled by contact method

	Fixed-line	%	Mobile	%
**Total numbers dialled**	**5390**	**100.0%**	**3697**	**100.0%**

**Eligible for study**	**190**	**3.5%**	**145**	**3.9%**

Completed interview	140	2.6%	128	3.5%

Personal refusal	19	0.4%	10	0.3%

Terminated/Incomplete interview	0	0.0%	0	0.0%

Unavailable for duration of survey	31	0.6%	7	0.2%

Ineligible (due to age, sex, language***)	**1935**	**35.9%**	**1215**	**32.9%**

**Unknown eligibility**	**785**	**14.6%**	**1309**	**35.4%**

No answer	386	7.2%	284	7.7%

Message left on recording device	331	6.1%	941	25.5%

Engaged	34	0.6%	70	1.9%

Refusal/Other	34	0.6%	14	0.4%

**Number invalid**	**2480**	**46.0%**	**1028**	**27.8%**

Business	284	5.3%	111	3.0%

Disconnected	1902	35.3%	897	24.3%

Fax/data line	294	5.5%	20	0.5%

Crude response rate* (%)	**73.7%**		**88.3%**	

Estimated response rate** (%)	**53.8%**		**45.0%**	

*completed interviews/eligible; see methods for detailed description

** completed interviews/(eligible + eligible/(eligible+ineligible) × unknown eligible); see methods for detailed description

***numbers with language difficulty comprised 15 of the ineligible fixed-line calls and 2 of the mobile calls

In univariate analyses, we found no significant difference between fixed-line and mobile participants in their responses to most of the main questionnaire items (Table 2). The exception was if the woman reported she was living with her parents (54% of fixed-line; 33% of mobile, p = 0.003). After adjusting for age, we found women contacted by mobile were significantly less likely to be living with their parents (OR 0.40, 95%CI 0.20-0.81, p = 0.01) and less likely to have reported ever having been pregnant (OR 0.44, 95%CI 0.24-0.81, p = 0.01).

**Table 2:** Response to main survey questions by contact method

	All		Contact Method				
			
			Fixed-line		Mobile		P-value
	**N**	**%**	**N**	**%**	**N**	**%**	

**Total participants**	**268**		**140**		**128**		

Age group*, years							0.7

18-20 yrs	47	17.5	27	19.3	20	15.6	

21-25 yrs	62	23.1	34	24.3	28	21.9	

26-30 yrs	91	34.0	43	30.7	48	37.5	

31-39 yrs	68	25.4	36	25.7	32	25.0	

State of residence*							0.9

New South Wales	89	33.2	47	33.6	42	32.8	

Victoria	69	25.7	34	24.3	35	27.3	

Queensland	55	20.5	29	20.7	26	20.3	

Other states	55	20.5	30	21.4	25	19.5	

Residence according to accessibility index**							0.2

Major city	167	63.5	82	59.4	85	68.0	

**Indigenous**							0.2

Yes	12	4.5	4	2.9	8	6.3	

**Country of birth**							0.6

Australia	192	71.6	102	72.9	90	70.3	

**Speaks a language other than English at home**							0.5

Yes	56	20.9	27	19.3	29	22.7	

**Highest education reached**							0.6

University degree or higher	92	34.5	50	36.0	42	32.8	

Currently living at home with parents***							0.003

Yes	83	43.7	53	54.1	30	32.6	

**Relationship status**							0.7

Single	87	32.5	44	31.4	43	33.6	

**Have a mobile phone**							

Yes	-	-	135	97.1	-	-	

**Have a landline at home**							

Yes	-	-	-	-	77	60.6	

**Received HPV vaccine**							0.6

Yes	137	51.7	74	53.2	63	50.0	

**Had diagnosis of genital warts**							0.9

Yes	17	6.5	9	6.6	8	6.4	

**Had Pap smear**							0.2

Yes	197	73.8	98	70.5	99	77.3	

**Tested for chlamydia**							0.3

Yes	84	33.2	48	36.1	36	30.0	

**Ever been pregnant**							0.07

Yes	122	45.5	71	50.7	51	39.8	

Percentages do not include missing values

*recruitment was stratified by these variables

** based on classification of 2006 ARIA+ score for residential postcode[14]

***only asked of women aged < 30 years

Excluding those who refused or could not answer the question, of women contacted by fixed-line, almost all reported having a mobile phone (97%, 135/139) while for those contacted by mobile phone, 39% (50/127) reported not having a "landline" at home. These 50 women, constituting a "mobile-only" population were a similar mean age to the 217 women who had a fixed-line at home (26.4 versus 27.1 years, p = 0.4). The mobile-only population were more likely to identify as Indigenous Australians (6/50 versus 6/217; age-adjusted OR 4.99, 95%CI 1.52-16.34, p = 0.008) and less likely to live with their parents (5/41 versus 78/149; age-adjusted OR 0.09, 95%CI 0.03-0.29, p < 0.001).

## Discussion

This is the first study we know of to compare random digit dialling mobile numbers to fixed-lines in the context of a general population survey of young people. We found that overall fewer numbers were dialled to achieve a completed interview by mobile phone than by fixed-line and while almost all participants contacted by fixed-line had a mobile (97%), a large proportion (39%) of those contacted by mobile did not have a fixed-line. We also found little difference between contact methods in terms of the response rates, and the characteristics of the women recruited.

Our data are comparable to other studies. The response rates achieved compared well to other recently conducted population health surveys where reported participation rates range from 26-42% [8-10]. Based on 2006 Australian census data in women of a similar age range to that targeted in our survey, 71% were Australian born, 3.1% were Indigenous and 73% lived in major cities [11]. Corresponding proportions in our combined sample across the two methods were 72%, 4.5% and 64%, although a higher percentage identified as Indigenous, particularly in the population contacted by mobile phone.

Our study was limited to young Australian women and as such the findings may not be applicable to other populations. Other studies have found that populations with access to mobile phones but not fixed-lines (i.e. "mobile-only") are younger and may undertake more risky behaviours such as smoking[5,12] although this is not consistently reported [13]. We found that after adjusting for age, comparing characteristics such as education and common health behaviours in this age range, there was no difference between the mobile-only population and other participants. Mobile-only women were more likely to identify as Indigenous and were less likely to live with their parents, but the modest sample size limits our ability to definitively comment on potential implications of these observations.

A major consideration before conducting a survey using random digit dialling mobile phones is the age of the target population. Our survey and others show that a substantial proportion of young people are only accessible through mobile phones[5,12] and that not surveying this mobile-only population may bias survey estimates [7]. The disadvantages of conducting surveys using mobiles include the potential increased call costs, particularly for longer surveys, and the inability to limit mobile calls to particular geographic regions within Australia. Future surveys should also include questions to allow weighting of sample populations for the ownership of more than one mobile or sharing of mobiles.

## Conclusions

In summary, our study demonstrates the feasibility of conducting a national population health survey by random digit dialling mobile phones. Studies like ours are important because if current trends in diminishing availability of fixed-lines continues,[5,7] particularly for researchers targeting young adults, surveys that rely on mobile phone contact will become a necessary survey tool.

## Competing interests

This study was funded by the Australian National Health and Medical Research Council (NHMRC) grant no. 568971 and the Victorian Cytology Service. BL and JB own shares in Telstra, an Australian telecommunications company. The other authors have no competing interests to declare.

## Authors' contributions

All authors were involved in the study conception and design, interpretation of data, and reviewed drafts of the manuscript. BL conducted the data analysis and wrote the initial draft. All authors read and approved the final manuscript.

## Pre-publication history

The pre-publication history for this paper can be accessed here:

http://www.biomedcentral.com/1471-2288/11/159/prepub

## Supplementary Material

Additional file 1 **Telephone survey questionnaire**. Questions that study participants were asked in the telephone survey. Click here for file
