# Supplementary material for: Mobile phones are a viable option for surveying young Australian women: a comparison of two telephone survey methods
Source: BMC Med Res Methodol. 2011 Nov 24;11:159. doi: 10.1186/1471-2288-11-159 (PMC3235070; doi:10.1186/1471-2288-11-159)
Supplement: Additional file 1 — Telephone survey questionnaire. Questions that study participants were asked in the telephone survey. [file 1471-2288-11-159-S1.DOC]

**TELEPHONE SURVEY**

| **Question No.** |  | **Response types** | **Next Question** |
| --- | --- | --- | --- |
| **INTRO DEM** | **We’ll start with a few general questions about yourself** |  | Go to D1 |
| D1 | What year were you born in? | Enter number (range to be specified)  OR refuse | If refuse go to D1A  Otherwise Go to D2 |
| D1A | Would it be easier for you to tell me which age group you are in? | - 20-24 years - 25-29 years - 30-34 years - 35-39 years - Refused | Go to D2 |
| D2 | What is your postcode of residence? | Enter number  OR refuse | If refuse go to D2A  Otherwise Go to D3 |
| D2A | Can you tell me what state or territory you live in? | - NSW - Victoria - Queensland - South Australia - Western Australia - Tasmania - ACT - NT - Refused | Go to D3 |
| D3 | What country were you born in? | Drop down list  OR refused | Go to D4 |
| D4 | Were you a permanent resident or citizen of Australia in 2007? (that is, eligible for Medicare) | - Yes - No - Unsure - Refused | Go to D4A |
| D4A | Have you been living in Australia between 2007 and now? | - Yes - No - Unsure - Refused | Go to D5 |
| D5 | Are you of Aboriginal or Torres Strait Islander origin? | - No - Yes-Aboriginal - Yes-Torres Strait Islander - Yes-both - Refused | Go to D6 |
| D6 | Do you speak a language other than English at home? | - Yes - No - Refused | Go to D7 |
| D7 | What is the highest educational qualification you have completed? | - No formal schooling - School or intermediate certificate - Technical or trade certificate - Higher secondary school/HSC/VCE/Leaving certificate - College certificate/diploma - University degree or higher - Refused | Go to D7A |
| D7A | Can you tell me what year you finished school (do not include University or college)? | Enter number (range to be specified)  OR refuse | If age<30 years or unknown go to D7B. Otherwise go to D8 |
| **D7B** | Do you currently live at home with one or both of your parents? | - Yes - No - Refused | Go to D8 |
| D8 | What is your current relationship status? | - Single (not in any type of relationship) - In a casual relationship or relationships - In a committed relationship, not living together - In a committed relationship, living together, not married - In a committed relationship, married - Refused | Go to D9 |
| **D9** | As we contacted you for this survey by landline and we are interested in finding out about whether it may be better to contact women for this survey by mobile, could I ask whether you have a mobile phone? | - Yes - No - Refused | Go to next section |
| **INTRO**  **REPRO** | **I now want to ask about some current issues affecting woman’s reproductive health** |  | Go to R1 |
| R1 | Have you had the HPV vaccine, which is also called the cervical cancer vaccine? | - Yes - No - Unsure | If yes go to R1AA  If no or unsure go to R2 |
| R1AA | Was the name of the vaccine you received? | - Gardasil - Cervarix - Unsure | Go to R1A |
| R1A | How many doses of the HPV vaccine have you received? | Enter number (range 1 to 6+)  OR unsure OR refuse | Go to R1B |
| R1B | Thinking about your first dose, where did you receive it? | - School - GP - Women’s health clinic - Sexual health clinic - Student health clinic - Other location (specify) - Can’t remember - Refuse | Go to R1C |
| R1C | In what month and year did you have your first HPV vaccine dose? | Enter month (range 1-12) OR Unsure  Enter year (2006 to current) OR Unsure | If month or year given go to R1D.  If Unsure and R1A>1 go to R1B1  If R1A=1, go to R1E |
| R1D | And can I just check, how certain are you that was the time you received it? | - Completely certain - Reasonably certain - Unsure - Refused | If R1A>1 go to R1B1  Otherwise go to R1E |
| R1B1 | Thinking about your second dose, where did you receive it? | - School - GP - Women’s health clinic - Sexual health clinic - Student health clinic - Other location (specify) - Can’t remember - Refuse | Go to R1C1 |
| R1C1 | In what month and year did you have your second HPV vaccine dose? | Enter month (range 1-12)  Enter year (2006 to current)  OR Unsure | If month or year given go to R1D1.  If Unsure and R1A>2 go to R1B2  If R1A=2, go to R1E |
| R1D1 | And can I just check, how certain are you that was the time you received it? | - Completely certain - Reasonably certain - Unsure - Refused | If R1A>2 go to R1B2  Otherwise go to R1E |
| R1B2 | Thinking about your third dose, where did you receive it? | - School - GP - Women’s health clinic - Sexual health clinic - Student health clinic - Other location (specify) - Can’t remember - Refuse | Go to R1C2 |
| R1C2 | In what month and year did you have your third HPV vaccine dose? | Enter month (range 1-12)  Enter year (2006 to current)  OR Unsure | If month or year given go to R1D2.  If Unsure go to R1H |
| R1D2 | And can I just check, how certain are you that was the time you received it? | - Completely certain - Reasonably certain - Unsure - Refused | Go to R1H |
| R1E | Are you planning to have any more HPV vaccine doses? | - Yes - No - Unsure | If yes go to R1F  If no or unsure go to R1G |
| R1F | What is the reason you haven’t received your next dose? (*If more than one reason ask for main reason and code that*) | - Not due yet - Forgot - Lack of time - Costs too much - Been away/travelling - Pregnancy - Vaccine was no longer available for free - Other (collect this) | Go to R2 |
| R1G | Why not/unsure? (*If more than one reason ask for main reason and code that*) | - Not sure of benefit of having any more doses - Advised by doctor not to have any more - I didn’t like my reaction to the vaccination - Worried about side effects - Costs too much - Vaccine was no longer available for free - Other (collect this) | Go to R2 |
| R1H | Did you receive a statement of completion in the mail from the National HPV vaccination program register? | - Yes - No - Unsure | If no or unsure go to R1J  If yes Go to R2 |
| R1J | Would you like me to give you the contact details of the Register so that you can contact them to get your statement? | - Yes – give phone no and email - No | Go to R2 |
| R2 | One of the diseases that the HPV vaccine can help prevent is genital warts. Have you ever had genital warts (*include anal warts if interviewer asked*)? | - Yes - No - Unsure - Wart virus on pap test/smear | If yes go to R2A, otherwise go to R3 |
| R2A | At what age were you first diagnosed? | Enter age (range <10 to current) | Go to R3 |
| R3 | A Pap smear or Pap test is a scraping of cells from the cervix collected by your doctor or nurse to help prevent cervical cancer. Have you ever had a Pap smear or Pap test? | - Yes - No - Unsure | If yes go to R3A,  If no go to R3A1  If unsure go to R4 |
| R3A | When was your last Pap test? | Enter year (range to be added) | Go to R3B |
| R3A1 | Do you intend to have one in the future? | - Yes - No - Unsure | If yes go to R3B  If no or unsure go to R3A2 |
| R3A2 | Why not? *(do not read out responses but code as participant provides)* | - Vaccinated, so don’t need it - Too old - Never had sex - Don’t want to - Married/monogamous relationship - Not sexually active now - Can’t say/refused - Other | Go to R4 |
| R3B | When are you planning to have your next Pap test? | - 1 year or less - 2 years - 3+ years - No longer need because of HPV vaccine - No longer need because of hysterectomy - Not sure - Other (collect this) | If R3B=1 year and R3A =2010 or 2011 go to R3C  Otherwise Go to R4 |
| R3C | Was your last Pap test abnormal? | - Yes - No - Unsure | Go to R4 |
| R4 | Chlamydia is the most commonly diagnosed sexually transmitted infection in Australia and it can affect a woman’s fertility. Have you ever been TESTED for Chlamydia (*from a swab or urine sample, do not include blood tests*)? | - Yes - No - Unsure | If yes go to R4A,  If unsure go to R4B  If no go to next section |
| R4A | How many times have you been tested? | Enter number  OR Unsure | Go to R4A1 |
| R4A1 | How old were you when you had your most recent test? | Enter age (range 10 to current)  OR Unsure | Go to R4B |
| R4B | Have you ever had a positive test for Chlamydia? | - Yes - No - Unsure | If yes go to R4C, otherwise go to next section |
| R4C | How many times have you had a positive Chlamydia test (give an estimate if you are not sure)? | Enter number  OR Unsure | Go to R4D |
| R4D | How old were you when you had your positive test for Chlamydia? (Ask for first positive test if more than one) | Enter age (range 10 to current)  Or Unsure | Go to R4G |
| R4G | Thinking about the time(s) you were diagnosed with Chlamydia, were all of your sexual partners notified and encouraged to have testing? | - Yes - No - Unsure | Go to next section |
| **INTRO**  **PREG** | Now a few questions about contraception and pregnancy. |  | Go to P1 if D8 indicates in a relationship, otherwise go to P2 |
| P1 | Are you using any form of contraception in your current relationship? (ask if indicated in a relationship) | - Yes - No - No, same sex relationship - Unsure | If yes, go to P1A  Otherwise go to P2 |
| P1A | What form of contraception is being used? (read out list) | - Contraceptive pill - Depo Provera injection - Implant (e.g. Implanon) - I have had a tubal ligation/hysterectomy - Condom - IUD - Safe period/natural family planning (rhythm method, Billings method, symptothermic, periodic abstinence) - Withdrawal (coitus interruptus**,** pulling out) - Douching (washing) - Diaphragm/cervical cap (prompt for foam/jelly use) - Spermicide foam or jelly - Female condom - Other nonprescribed - Don’t know/Can’t remember - Other (collect free text) - Refused | Go to P2 |
| P2 | Have you ever been pregnant? | - Yes - No - Unsure - Refused | If yes go to P2A  Otherwise go to P3 |
| P2A | Have you ever had a miscarriage? | - Yes - No - Unsure - Refused | If yes go to P2B, if no go to P2C |
| P2B | Could you please tell me how many? | Enter number   - OR Refuse | Go to P2C |
| P2C | Have you ever had a termination of pregnancy? (an abortion) | - Yes - No - Unsure - Refuse | If yes go to P2D  If no go to P3 |
| P2D | Could you please tell me how many? | Enter number  OR Refuse | Go to P3 |
| P3 | Have you ever experienced difficulties trying to get pregnant? | - Yes - No - Unsure, not tried - Refuse | If yes go to P3A  Otherwise if R1=yes or unsure (received HPV vaccine or unsure) & age<30 years, go to validation section; if R1=no then go to end |
| P3A | Have you ever had treatment to help you get pregnant? | - Yes - No - Unsure - Refuse | If yes go to P3B  Otherwise if R1=yes or Unsure (received HPV vaccine or unsure) & age<30 years, go to validation section; if R1=no then go to end |
| P3B | What sort of treatment have you had? (can select more than one) | - IVF (in vitro fertilization) - Surgery (e.g. endometriosis surgery) - Fertility hormones (e.g. Clomid) - Other - Refuse | If R1=yes or unsure (received HPV vaccine or unsure) & age<30 years, go to validation section; if R1=no then go to end |
| **VALIDATION SECTION** | Just one final question regarding the HPV vaccine, |  | Go to V1 if R1=yes  Go to V2 if R1=unsure |
| V1 | Earlier you said that you had received the vaccine. We would like to check the vaccine doses you remember receiving with the National Register and if necessary, with your doctor. This will help us understand how well people can remember what vaccines they have received. Would it be ok for us to send you a form to sign to allow us to do this? | - Yes – *enter name and mailing details* - No | Go to end interview |
| V2 | You said earlier you were not sure whether you have been vaccinated or not. We would like to check the national HPV vaccination register to see if there are any doses recorded for you. This will help us understand how well people can remember what vaccines they have received. Would it be ok for us to send you a form to sign to allow us to do this? | - Yes – *enter name and mailing details* - No | Go to end interview |

That’s the end of the study questions. Thank you very much for your help.

**FOR MOBILE RANDOM DIGIT DIALLING**

The questionnaire will be exactly the same as for the landline random digit dialling except for question D9 which for the mobile interview will be replaced by the following question.

| **Question No.** |  | **Response types** | **Next Question** |
| --- | --- | --- | --- |
| **D9**  **(mobile only)** | As we contacted you for this survey by your mobile phone, and we are interested in finding out if mobile users also have landlines, could I ask whether you have a landline telephone at home? | - Yes - No - Refused | Go to next section |
